# Severe Anemia With Intraosseous-Intramuscular Hemorrhage in a Patient With Kit-C-Negative Systemic Mastocytosis

**DOI:** 10.7759/cureus.84798

**Published:** 2025-05-25

**Authors:** Natalia Fongrat, Anjali Davichan, Christopher Vaughn, Sagah Ahmed, Andrew Mangano

**Affiliations:** 1 Internal Medicine, Mary Washington Healthcare, Fredericksburg, USA; 2 Hematology and Oncology, Mary Washington Healthcare, Fredericksburg, USA

**Keywords:** ghost cell, intramuscular hemorrhage, intraosseous hemorrhages, kit-c negative, systemic mastocytosis

## Abstract

Systemic mastocytosis (SM) is a rare hematologic disorder characterized by clonal proliferation of mast cells in various tissues, often presenting with skin lesions, gastrointestinal symptoms, or anaphylaxis. However, atypical presentations lacking these classical features may delay diagnosis and complicate management. We report the case of a 42-year-old woman who presented with progressive anemia, recurrent spontaneous intramuscular and intraosseous hemorrhages, and severe, unexplained pain. Notably, she lacked cutaneous signs or known allergic triggers. Extensive laboratory and imaging workups ruled out common hematologic and autoimmune causes. Bone marrow biopsy ultimately revealed multifocal clusters of atypical mast cells consistent with SM, despite the absence of the KIT D816V mutation. This case underscores the protean manifestations of SM and the need to maintain a high index of suspicion in patients with unexplained cytopenia and bleeding. It also highlights the role of bone marrow evaluation in the diagnostic pathway, even when classic clinical features are absent. Timely recognition of atypical SM can guide appropriate management and improve patient outcomes.

## Introduction

Systemic mastocytosis (SM) is a rare hematologic disorder characterized by the clonal proliferation and accumulation of mast cells, i.e., granulated immune cells that play a key role in allergic reactions and innate immunity, in various tissues, including the bone marrow, skin, gastrointestinal tract, liver, and spleen [1]. The disease is primarily driven by activating mutations in the KIT proto-oncogene, which encodes a receptor tyrosine kinase essential for mast cell development and function. The most common mutation, KIT D816V, results in constitutive activation of the receptor and is found in over 80% of cases [2]. However, a subset of patients lacks detectable KIT mutations, known as KIT-negative SM, which presents diagnostic challenges and necessitates reliance on morphologic, immunophenotypic, and clinical criteria [3].

The clinical manifestations of SM are highly heterogeneous, reflecting both the extent of mast cell infiltration in various organs and the systemic effects of mast cell degranulation. Symptoms may range from indolent disease with minimal clinical impact to aggressive subtypes associated with multi-organ dysfunction and poor prognosis [1]. Classical symptoms, such as flushing, pruritus, abdominal cramping, diarrhea, and anaphylaxis, are primarily mediated by the release of mast cell-derived mediators like histamine, tryptase, and prostaglandins [4]. By contrast, hematologic abnormalities, including anemia, thrombocytopenia, and coagulopathy, are less frequently reported and may be underrecognized. In some patients, profound anemia or spontaneous bleeding may be the initial or predominant clinical feature, obscuring the diagnosis and delaying appropriate evaluation [3].

The absence of KIT mutations in certain cases further complicates diagnosis and underscores the need for heightened clinical suspicion. In patients presenting with unexplained cytopenias, bleeding diathesis, or bone marrow abnormalities, SM should be considered, particularly when other causes have been excluded. A comprehensive diagnostic approach, including bone marrow biopsy with immunohistochemistry, flow cytometry to detect aberrant mast cell markers (e.g., CD25, CD2), measurement of serum tryptase levels, and molecular analysis for KIT and non-KIT mutations, is essential for confirming the diagnosis [1].

This report aims to explore the hematologic manifestations of SM, with particular attention to mast cell-driven bleeding complications and the diagnostic nuances in KIT-negative cases. By highlighting an atypical presentation, this study emphasizes the importance of recognizing SM as a potential underlying cause of otherwise unexplained hematologic abnormalities.

## Case presentation

The patient is a 42-year-old female with a past medical history of asthma, Roux-en-Y gastric bypass (performed in 2020), and depression who presented with abdominal pain and severe anemia.

Initial presentation and diagnostic workup

**Figure 1:**
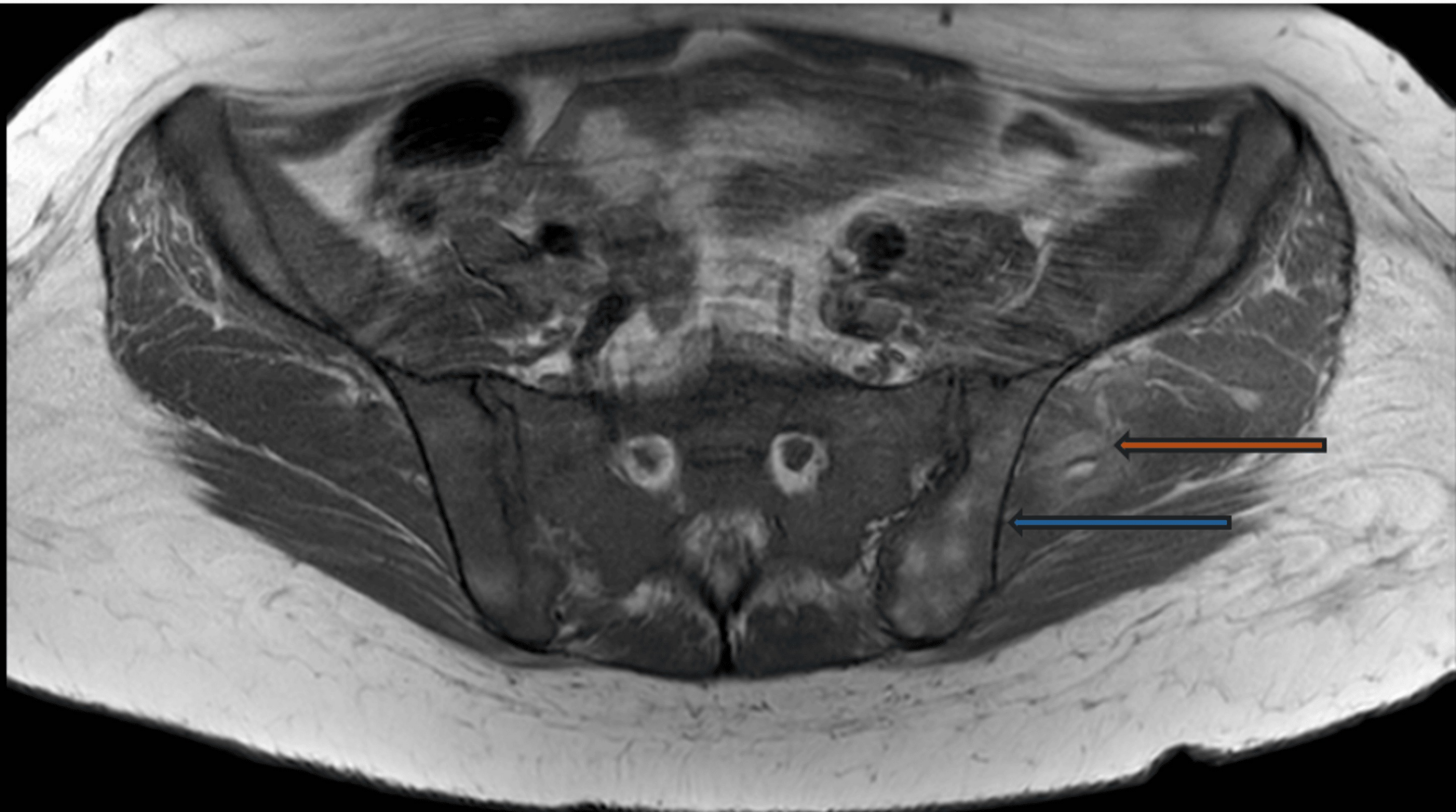
MRI abdomen and pelvis with and without IV contrast. The blue arrow showing multifocal intraosseous and orange arrow showing intramuscular hemorrhage in the left iliopsoas and gluteal regions.

Five months before her diagnosis, the patient developed bilateral ribcage pain and dyspnea, leading to an emergency department (ED) visit, where she was diagnosed with pneumonia and prescribed antibiotics. She later developed left breast bruising and swelling and was treated empirically for soft tissue infection. Around the same time, she experienced persistent low back and buttock pain, unrelieved by steroids, followed by acute left pelvic pain radiating to her thigh, prompting another ED visit. At that time, her hemoglobin had dropped to 5.7 grams per deciliter (g/dL) from a baseline of ~10 g/dL. Abdominal and pelvic magnetic resonance imaging (MRI) with and without intravenous (IV) contrast showed hepatosplenomegaly and nontraumatic intraosseous and intramuscular hemorrhage in the left iliopsoas and gluteal regions (Figure 1). 

The differential diagnosis included infection and noninfectious inflammatory conditions, such as hemorrhagic myonecrosis and hemorrhagic bony infarcts. The patient received three units of packed red blood cells (pRBC) and was empirically treated for infectious myositis. Laboratory findings included a low Immunoglobulin G (IgG) level of 375 milligrams per deciliter (reference range: 767-1590 mg/dL), mildly elevated erythrocyte sedimentation rate (ESR), and C-reactive protein (CRP). Extensive infectious testing, including blood cultures, human immunodeficiency virus (HIV) PCR, hepatitis panel, Lyme immunoglobulin M and G (IgM, IgG), and *Treponema pallidum* antibodies, was all negative. An esophagogastroduodenoscopy (EGD) was performed, and gastrojejunal anastomosis with ulceration was identified. Biopsy results showed no evidence of malignancy or *Helicobacter pylori*. The ulcer was not considered the likely culprit of her anemia, and a bone marrow biopsy showed necrotic marrow with minimal viable cellularity. Coagulation studies revealed a slightly prolonged prothrombin time. Hemolysis markers were notable for elevated lactate dehydrogenase and an increased reticulocyte percentage and immature reticulocyte fraction, despite a decreased absolute reticulocyte count. Total bilirubin was mildly elevated, while direct bilirubin remained within the normal range (Table 1). Additional testing, including partial thromboplastin time, comprehensive metabolic panel, antinuclear antibodies, creatine kinase, vitamin B12, folate, rheumatoid factor, antineutrophil cytoplasmic antibodies (p-ANCA, c-ANCA), alpha-fetoprotein, copper levels, lupus anticoagulant, anti-Jo antibodies, ferritin, haptoglobin, and a direct Coombs test, was unremarkable. Serum and urine protein electrophoresis demonstrated an acute-phase reactant response without evidence of malignancy. The patient's hematologic parameters remained within normal limits, allowing for discharge with scheduled follow-up appointments with hematology as an outpatient.

**Table 1:** Laboratory findings summary

Laboratory parameters	Value	Reference range
Prothrombin time (PT)	11.9 sec	9.5-11.5 sec
Lactate dehydrogenase (LDH)	489 U/L	100-190 U/L
Absolute reticulocyte count	0.09	0.5-2.5
Reticulocyte percentage	6.0%	0.5-2.5%
Immature reticulocyte fraction	37.8%	<15%
Total bilirubin	1.4 mg/dL	0.1-1.2 mg/dL
Direct bilirubin	0.3 mg/dL	0.0-0.3 mg/dL

Progressive symptoms and hematologic evaluation

Two weeks later, the patient was readmitted with hemoptysis and left ear pain. Examination revealed active bleeding in the left middle ear, which was draining through the Eustachian tube into the oropharynx, leading to expectoration of blood. Severe anemia returned with a hemoglobin level of 6 g/dL. She received two units of pPRBCs, after which her hemoglobin stabilized at 8.7 g/dL. A repeat ENT evaluation noted improvement in hemotympanum. MRI of the temporal bones and a CT scan of the neck to assess for paraganglioma; however, the patient declined these studies. The Hematology team was consulted and advised to start intravenous aminocaproic acid, followed by a transition to an oral formulation. However, due to difficulty swallowing large pills, the patient declined the oral medication and was instead switched to tranexamic acid (TXA), which she tolerated well. For discharge planning, Hematology recommended continuing TXA for an additional three days. Given the patient’s occasional complaints of flushing, a tryptase level was obtained and found to be markedly elevated at 1,191 micrograms per liter (mcg/L) (normal range <11 mcg/L), raising suspicion for mastocytosis. Testing for KIT mutation and beta-glucosidases was ordered to assess for mastocytosis and Gaucher’s disease, respectively, both of which can manifest with bleeding, osteonecrosis, and hepatosplenomegaly were negative. The patient was discharged home with close follow-up.

The patient returned to the emergency department a week later with chest pain and intermittent, patchy skin rashes along with generalized fatigue. A computed tomography angiogram (CTA) chest showed no pulmonary embolism (PE), a slight increase in bilateral pleural effusions with bibasilar atelectasis versus pneumonitis, a new indeterminate inflammatory process in the right chest wall/axilla, and a stable anterior mediastinal nodule. The initial bone marrow biopsy from the previous admission returned and showed significant abnormalities, including extensive necrosis, raising suspicion for mastocytosis. A right axilla biopsy and repeat bone marrow biopsy were done. Axilla biopsy indicated mastocytosis. A concurrent second bone marrow biopsy showed aggregates of necrotic ghost cells, with flow cytometry showing no evidence of lymphoproliferative disorder, acute leukemia, or increased blasts. Cytogenetics revealed a normal karyotype. Additional testing revealed elevated factor VIII level, ferritin, and LDH (Table 2). Flow cytometry demonstrated rare CD117-bright events with no significant blast population and polytypic B cells with no aberrant immunophenotype on T cells. A minute CD117-bright population was identified, which represented <0.1% of the total events. This population appeared positive for CD25. In the appropriate clinical context, this population could indicate circulating mast cells, but the limited number of events prevented definitive characterization. Given these findings, and with her ongoing symptoms, she was started on prednisone 40 milligrams (mg) daily with a planned taper of 10 mg per week. 

**Table 2:** Hematologic laboratory results

Hematologic labs	Lab result	Reference range and units
Prothrombin time	12.7	9.0-13.0 sec
Prothrombin time INR	1.1	0.8-1.2
Partial thromboplastin time	28.0	25.0-38.5 sec
Ferritin	568	5-200 ng/mL
Fibrinogen	368	150-400 mg/dL
Iron	107	50-170 ug/dL
Transferrin	208	200-340 mg/dL
Haptoglobin	146	30-200 mg/dL
Lactate dehydrogenase	281	125-250 U/L
Folate, serum	8.6	7.0-31.0 mg/mL
Factor XIII antigen	116	50-150%
Factor VIII assay	273	50-150%
Von Willebrand factor AG	211	66-176%
Von Willebrand factor activity	192	50% - von Willebrand Disease unlikely; if strong clinical suspicion, retest at another time

Acute pain crisis and confirmation of mastocytosis

**Figure 2:**
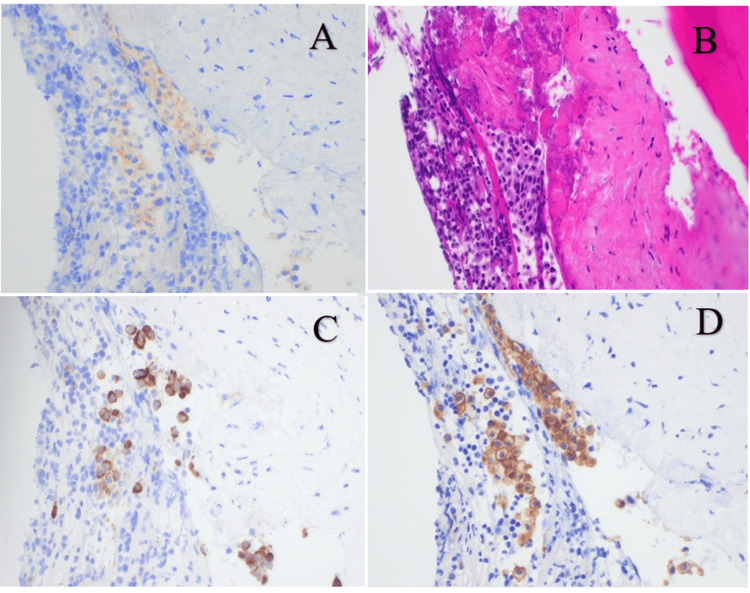
Bone marrow biopsy findings demonstrating mast cell involvement. (A) Immunohistochemical staining for CD25 shows scattered mast cells with focal positive staining, indicating aberrant expression (brown). (B) Hematoxylin and eosin (H&E) staining reveals a cellular focus of mast cells with spindled morphology infiltrating the marrow. (C) Tryptase staining highlights numerous positively stained mast cells, confirming their identity (brown cytoplasmic staining). (D) CD117 (c-KIT) immunohistochemistry shows strong membranous and cytoplasmic positivity in clustered mast cells, consistent with mast cell proliferation.

A few weeks later, while off steroids, the patient underwent a final bone marrow biopsy, which demonstrated a cellular marrow with trilineage hematopoiesis and no increase in blasts, indicating preserved hematopoietic function (Table 3). Scattered mast cells were identified, including a rare focus of abnormal spindled CD25-positive mast cells, an immunophenotypic feature supportive of SM (Figure 2). Peripheral blood studies at that time revealed macrocytic anemia and absolute lymphopenia, consistent with ongoing hematologic involvement. These findings, when integrated with the elevated serum tryptase level and the morphologic features noted in the marrow, supported the diagnosis of SM and guided subsequent treatment decisions. Following the confirmed diagnosis, avapritinib was initiated. The patient has been maintained on montelukast, prednisone, and avapritinib. Treatment response is being monitored through serial serum tryptase levels and ongoing outpatient follow-up with hematology.

**Table 3:** Peripheral blood and bone marrow aspirate

Bone marrow aspirate		
Cell type	Value %	Normal range %
Blasts	2.5	0-3
Promyelocytes	2.5	1-8
Myelocytes	11	5-19
Metamyelocytes	12	13-22
Band neutrophils	14	12-34
Erythroblasts (NRBCs)	34.5	7-32
Hematologic findings		
Laboratory parameters	Value	Reference range
WBC	4.24 k/uL	4.0-11k/uL
Hemoglobin	9.0 g/dL	12.0-15.0 g/dL
Hematocrit	28.2%	35.5-44.9%
MCV	98.6 L	80-100 L
Platelets	289 k/uL	150-450 k/uL
Peripheral differential		
Cell type	Value %	Normal Range %
Blasts		0%
Promyelocytes		0%
Myelocytes	0.9	0%
Metamyelocytes	0.9	0%
Band neutrophils		0-5%
Seg. neutrophils	73.0	40-70%
Eosinophils	1.7	0-6%
Basophils		0-1%
Monocytes	4.3	2-10%
Lymphocytes	15.7	20-40%
Atypical lymph	3.5	<5%
Other cells		-
Erythroblasts (NRBCs)		0%

## Discussion

Mastocytosis is a rare condition characterized by excessive mast cell accumulation, primarily affecting the skin and bone marrow. Patients with unexplained anaphylaxis, recurrent flushing, chronic skin lesions such as urticaria pigmentosa, or persistently elevated serum tryptase levels should be evaluated for mastocytosis. Additional indications include unexplained cytopenia; osteopenia; chronic gastrointestinal symptoms like diarrhea, nausea, and abdominal pain; or unexplained bleeding. Individuals with a family history of mast cell disorders, resistant allergic conditions, or multi-organ involvement without a clear etiology should also be considered for further testing [5,6].

The World Health Organization (WHO) defines SM based on specific diagnostic criteria, including dense mast cell infiltrates in extracutaneous organs, marrow, or blood, as well as minor criteria such as atypical mast cell morphology, KIT D816V mutations, CD25 expression, and persistently elevated serum tryptase levels. Bone marrow biopsy is essential for detecting these abnormalities and confirming the diagnosis [7].

Diagnosing SM in patients who lack the KIT D816V mutation is particularly challenging. The KIT D816V mutation is identified in over 80% of SM cases, making it a key diagnostic marker. However, some patients exhibiting clinical and histopathologic features suggestive of SM test negative for this mutation, complicating the diagnostic process [8].

In KIT D816V-negative SM, the absence of this genetic marker necessitates a more extensive diagnostic approach, incorporating morphology, immunophenotyping, and serum biomarkers. Bone marrow biopsy remains essential, particularly in identifying increased mast cell burden, spindle-shaped mast cells, and aberrant CD25 expression. However, in some cases, mast cell infiltration may be minimal or obscured by extensive fibrosis and necrosis, leading to sampling inconsistencies and potential underdiagnosis [5,8].

Flow cytometry can help detect abnormal mast cells, even when histopathology is inconclusive. In addition, elevated serum tryptase levels serve as a useful diagnostic marker but may not always be present, especially in early or indolent SM cases. To further investigate the underlying pathology, next-generation sequencing (NGS) or whole-exome sequencing can identify alternative genetic mutations such as JAK2, TET2, SRSF2, ASXL1, or RUNX1, which are sometimes associated with KIT-negative SM, particularly in advanced or aggressive forms of the disease [9].

Due to these diagnostic challenges, patients with KIT-negative SM often require multiple bone marrow biopsies and serial assessments over time to reach a definitive diagnosis. Importantly, KIT negativity does not rule out SM, but rather suggests greater molecular and genetic variability, which may influence disease progression and treatment options [5,8].

Bleeding in mastocytosis results from multiple factors, including heparin release, platelet dysfunction, coagulopathy, gastrointestinal bleeding, and vascular fragility [10]. Excess heparin disrupts clot formation, while histamine and prostanoids impair platelet aggregation [11]. Our patient’s bleeding was likely due to a combination of coagulation abnormalities, mast cell infiltration, and chronic inflammation. Mast cell-derived mediators, including elevated factor VIII (as seen in our case) and von Willebrand factor, contribute to dysregulated coagulation. In addition, tryptase and chymase degrade fibrinogen and alter thrombin-induced clotting, further impairing hemostasis. Mast cell infiltration can compromise mucosal integrity, increasing the risk of gastrointestinal bleeding, while chronic inflammation weakens vascular walls, making them more prone to spontaneous rupture and hemorrhage [6,10,11].

A notable finding in this case was the presence of ghost cells in bone marrow, indicative of lysed or degenerated cells. In mastocytosis, these structures suggest mast cell degranulation, leading to the release of histamine, heparin, and other bioactive mediators. This process contributes to tissue damage, fibrosis, and, in advanced cases, bone marrow necrosis [8,9].

Management of SM depends on its subtype, symptom severity, and organ involvement, with treatment aimed at controlling symptoms, reducing mast cell burden, and preventing complications. Antihistamines, mast cell stabilizers, and cytoreductive therapies such as tyrosine kinase inhibitors (e.g., midostaurin, avapritinib) are key components of therapy, while chemotherapy is reserved for aggressive forms like mast cell leukemia. Bisphosphonates help manage osteoporosis and bone pain, and avoiding triggers such as heat, alcohol, and certain medications is crucial. KIT-negative mastocytosis, as in our case, may require a tailored approach, but the primary goal remains preventing severe reactions and improving quality of life [1,5].

Regular monitoring with serum tryptase measurement, organ function assessment, and genetic testing is essential for tracking disease progression [12]. Management often involves a multidisciplinary approach, including hematology, oncology, and allergy/immunology specialists. Given the underdiagnosed nature of mastocytosis, heightened clinical awareness is necessary, especially in patients with unexplained allergic reactions, hematologic abnormalities, and bleeding disorders. The identification of ghost cells in bone marrow may serve as an important diagnostic clue, highlighting increased mast cell activity. A thorough evaluation, including bone marrow biopsy, serum tryptase testing, and KIT mutation analysis, is crucial for accurate diagnosis and optimal management [13].

## Conclusions

Mastocytosis is a rare and underdiagnosed disorder characterized by abnormal mast cell accumulation with systemic involvement. The presence of ghost cells may indicate mast cell degranulation and disease progression. Patients with unexplained allergic reactions, bleeding tendencies, or persistent gastrointestinal symptoms should be evaluated.

Early recognition and comprehensive diagnostic testing, including bone marrow biopsy, serum tryptase measurement, and KIT mutation analysis, are essential. Diagnosing KIT-negative SM remains challenging, requiring a multimodal approach. Advances in molecular research and diagnostic techniques may improve early detection and guide individualized treatment strategies.
